# Deleterious Effect of High-Fat Diet on Skeletal Muscle Performance Is Prevented by High-Protein Intake in Adult Rats but Not in Old Rats

**DOI:** 10.3389/fphys.2021.749049

**Published:** 2022-01-17

**Authors:** Eleonora Poggiogalle, Fanny Rossignon, Aude Carayon, Fréderic Capel, Jean-Paul Rigaudière, Sarah De Saint Vincent, Olivier Le-Bacquer, Jérôme Salles, Christophe Giraudet, Véronique Patrac, Patrice Lebecque, Stéphane Walrand, Yves Boirie, Vincent Martin, Christelle Guillet

**Affiliations:** ^1^Medical Pathophysiology, Food Science and Endocrinology Section, Department of Experimental Medicine, Sapienza University of Rome, Rome, Italy; ^2^INRA, UNH, Unité de Nutrition Humaine, CRNH Auvergne, Clermont Auvergne University, Clermont-Ferrand, France; ^3^AME2P, Université Clermont Auvergne, Clermont-Ferrand, France; ^4^Institut Universitaire de France, Paris, France

**Keywords:** sarcopenic obesity, dynapenia, muscle lipid content, muscle function, aging

## Abstract

The phenotype of sarcopenic obesity is frequently associated with impaired muscle strength and performance. Ectopic lipid deposition may interfere with muscle anabolic response especially during aging. Evidence is scarce concerning the potential interplay among aging and nutrient imbalance on skeletal muscle functionality. The objective of the present study was to investigate the impact of protein intake in the context of an obesogenic diet on skeletal muscle functional properties and intramuscular lipid infiltration. Two groups of forty-two adult and thirty-seven old male Wistar rats were randomly divided into four groups: isocaloric standard diet (12% protein, 14% lipid, as ST12); isocaloric standard (high-protein) diet (25% protein, 14% lipid, ST25); hypercaloric high-fat (normal-protein) diet (12% protein, 45% lipid, HF12); and hypercaloric high-fat (high-protein) diet (25% protein, 45% lipid, HF25). The nutritional intervention lasted 10 weeks. Total body composition was measured through Echo-MRI. Lipids were extracted from tibialis anterior muscle and analyzed by gas-liquid chromatography. The functional properties of the plantarflexor muscles were evaluated *in vivo* on an isokinetic dynamometer. Maximal torque was assessed from the torque-frequency relationship in isometric condition and maximal power was evaluated from the torque-velocity relationship in concentric condition. In adult rats high-protein intake combined with high-fat diet determined a lower decrease in relative isometric torque, normalized to either FFM or body weight, compared with adult rats fed a high-fat normal-protein diet. High-fat diet was also detrimental to relative muscle power, as normalized to body weight, that decreased to a larger extent in adult rats fed a high-fat normal-protein diet than their counterparts fed a normal-fat, high-protein diet. The effect of high-fat diet observed in adults, with the enhanced protein intake (25%) conferring some kind of protection against the negative effects of HFD, may be linked to the reduced intramuscular fat in this group, which may have contributed to preserve, at least partly, the contractile properties. A potential role for high-protein diet in preventing ectopic lipid deposition needs to be explored in future research. Detrimental effects of high- fat diet on skeletal muscle performance are mitigated by high- protein intake in adult rats but not in old rats.

## Introduction

Sarcopenia is the progressive and generalized loss of skeletal muscle mass and function, frequently reported in older people and in patients with chronic diseases (Cruz-Jentoft et al., 2014; Tournadre et al., 2019). The maintenance of skeletal muscle mass results from a balance between muscle protein synthesis and breakdown (Andreou and Tavernarakis, 2010). During aging or chronic diseases, one of the principal determinants of skeletal muscle protein loss is a muscle “anabolic resistance” (Paulussen et al., 2021). More precisely, this metabolic alteration concerns a reduced sensitivity of muscle protein synthesis response to normal anabolic stimuli, such as nutrients and/or exercise (Guillet et al., 2004; Dardevet et al., 2012; Beals et al., 2018). This weaker or lack of anabolic response induces an imbalance in skeletal muscle protein turnover in favor of protein degradation leading progressively to muscle protein losses. An appropriate intake of dietary protein is also pivotal to maintain muscle mass or prevent rapid muscle mass loss (Bauer et al., 2013). In older individuals, a reduction in dietary protein digestion and absorption kinetics after protein ingestion has been observed and might contribute to age-related anabolic resistance of muscle protein synthesis (Wall et al., 2015; Gorissen et al., 2020). This age-related dysregulation of protein metabolism and other specificities of advancing age, influence the requirement of older people for dietary protein. Consequently, the recommended dietary allowance (RDA) for protein (0.8 g/kg/day) for adult people might not be sufficient for preserving muscle mass and quality on a long-term basis in older age (Bauer et al., 2013). According to the experts of PROT-AGE study, older people should consume an average daily protein intake of 1.0–1.2 and 1.2–1.5 g/kg/day in disease states. In addition, the amount of protein consumed per meal is important for muscle mass and function (Bauer et al., 2013). Indeed, consuming frequent meals containing 30–45 g of protein is associated with greater leg lean mass and knee extensor muscle strength (Loenneke et al., 2016). This nutritional strategy was also associated with higher appendicular lean body mass (an index of muscle mass) in both older men and women at baseline and after a 2-year follow-up period (Farsijani et al., 2016). Moreover, elevated intake of whey protein was also efficient on the stimulation of muscle protein synthesis in healthy, older men (Pennings et al., 2012), potentially overcoming muscle anabolic resistance in this population. The postprandial muscle anabolic resistance observed in healthy elderly people is exacerbated in obese elderly people with reduced physical activity levels (Smeuninx et al., 2017). In general, obesity and high-fat, high-calorie diets are accompanied by anabolic resistance at the muscle and whole-body level in rodents as in humans (Chevalier et al., 2005; Anderson et al., 2008; Guillet et al., 2009; Beals et al., 2018). In models of obesity and aging, the decreased muscle protein anabolism was associated with ectopic accumulation of specific fatty acids and derivatives (such as ceramides) involved in lipotoxicity within skeletal muscle (i.e., myosteatosis) (Tardif et al., 2014). This ectopic infiltration within skeletal muscle is mainly due to the decreased buffering capacity of adipose tissue. This involves a reorientation of excess circulating lipids toward storage in peripheral non-adipose tissues such as the liver or skeletal muscle. In addition, age and obesity are associated with reduced mitochondrial fatty acid oxidation capacities which could induce or amplify ectopic lipid accumulation in the skeletal muscle (Tardif et al., 2014; Capel et al., 2016). *In vitro* studies have confirmed this phenomenon by showing that myotubes exposed to (i) specific fatty acids (palmitic acid) or (ii) conditioned media from insulin resistant adipocytes produced deleterious lipid mediators (ceramides and diacylglycerols) associated with a decrease in muscle insulin sensitivity and a reduced rate of protein synthesis (Tardif et al., 2014; Capel et al., 2016). Animal studies clearly indicated that old rats and old obese rats were more prone to ectopic muscle lipid accumulation than young counterparts, leading to decreased muscle protein anabolism (Masgrau et al., 2012; Tardif et al., 2014). In young obese mice and old non-obese mice an accumulation of deleterious lipid derivatives (DAG and ceramides) was observed in skeletal muscles, associated with not only a decrease in the anabolic response muscle but also a reduction in muscle strength (Rivas et al., 2016). In addition, muscle fat infiltration affects skeletal muscle quality, as evidenced by a lower muscle strength and poor physical performance (Visser et al., 2002; Delmonico et al., 2009).

Since elevated protein intake can overcome anabolic resistance related to age, we hypothesized that a higher intake of dietary protein might also improve muscle quality within the context of high-fat diet. The objectives of the study were to determine the impact of protein intake in the context of an obesogenic diet on muscle function and the benefit of increasing protein intake to counteract the muscle alterations induced by obesity and aging.

## Materials and Methods

### Animals and Experimental Procedures

Experiments were conducted according to guidelines for the care and use of animals, and approved by the local Ethical Committee for animal experimentation. Forty-seven adult and thirty-eight old male Wistar rats (CERJ Janvier, Le Genest-Saint-Isle, France) – aged 9 and 22 months, respectively – were individually housed with free access to water under standard conditions (controlled room temperature 20–22°C, inverted 12:12 h light-dark cycle). The study was preceded by a run-in period of 1 week in which animals were fed powdered food. The amount of food during the study was provided on a weekly basis, and the same weekly frequency was used to assess the amount of powder ingested. After 1-week acclimatization, animals were randomly divided into four groups, according to body weight, body fat, and fat-free mass assessed by Echo-MRI^®^ (EchoMRI^®^ 900, Houston, TX, United States) at baseline, as follows: isocaloric (3.9 kcal/g) standard (normal-protein) diet (12% protein, 14% lipid, and 74% carbohydrate) (ST12); isocaloric (3.9 kcal/g) standard (high-protein) diet: (25% protein, 14% lipid, and 61% carbohydrate) (ST25); hypercaloric (4.8 kcal/g) high-fat (normal-protein) diet: (12% protein, 45% lipid, and 43% carbohydrate) (HF12); and hypercaloric (4.8 kcal/g) high-fat (high-protein) diet: (25% protein, 45% lipid, and 30% carbohydrate) (HF25). The duration of nutritional intervention was 10 weeks. The two high-fat diets had been originally designed as hypercaloric. Rats in the high-energy high-fat diets self-limited their dietary intake, with no differences in terms of energy intake, leading to a macronutrient manipulation study. Dietary composition is summarized in Supplementary Table 1. Casein was the source of protein in all diets. The source of fat was represented by soy oil (14% of the total energy intake- TEI, in the standard diets) and a mixture of soy oil (7% TEI) and lard (38% TEI) in the high-fat diets. Diets were prepared specifically for the present study in the Unit of Provision of Experimental Foods (Unité de Préparation des Aliments Expérimentaux, INRA, Jouy-en-Josas, France). The group distribution of rats was the following: adult rats: ST12 (*n* = 12); ST25 (*n* = 12); HF12 (*n* = 12); HF25 (*n* = 11). Old rats: ST12 (*n* = 11); ST25 (*n* = 11); HF12 (*n* = 9); HF25 (*n* = 7).

### Body Weight and Body Composition

Body weight was measured on a weekly basis from baseline to week 10. Total body composition [two compartments: fat mass (FM) and fat-free mass (FFM)] was measured at baseline (time 0: “T0”), after 5 weeks (time 1: “T1”), and at week 10 (time 2: “T2”), at the end of the study, through Echo-MRI (EchoMRI^®^ 900, 3-in-1 composition analyzer Echo Medical Systems, Houston, TX, United States) (Wall et al., 2015). This type of analyzers deliver precise body composition measurements of fat, lean, free water, and total water masses in lived animals.

### Glucose Homeostasis and Insulin Resistance

Blood was collected in the fasting state from the aorta artery after rats were sacrificed. Glucose concentrations were determined by using Konelab 20 (Thermo Electron Corporation) and Konelab system reagents (Thermo Fisher Scientific, Vantaa, Finland). Plasma insulin concentrations were measured using an ELISA kit (Millipore Corporate Headquarters, Billerica, MA, United States). The fasting glucose-to-insulin ratio (FGIR) was calculated (Bligh and Dyer, 1959; Cacho et al., 2008; Bowe et al., 2014).

### Intramuscular Lipid Content

Lipids were extracted from tibialis anterior muscle according to Bligh and Dyer (1959) in the presence of the internal standards. Triacylglycerols (TAGs) were analyzed by gas-liquid chromatography on a FOCUS Thermo Electron system using a Zebron-1 Phenomenex fused silica capillary column (5 m 9 0.32 mm i.d., 0.50 ml film thickness).

### Evaluation of Skeletal Muscle Functional Properties

The functional properties of the plantarflexor muscles were evaluated *in vivo* on an isokinetic dynamometer specially designed for rats (806D, Aurora Scientific, Canada). The testing protocol was based on protocols used by previous studies (Warren et al., 2004; Martin et al., 2013).

Rats were maintained anesthetized with continuous isoflurane inhalation (2%). During the testing procedures, the rat laid supine on a heating plate with the right foot attached to a footplate connected to a dual mode servomotor (305C-LR, Aurora Scientific, Canada). The knee was clamped in place such that the knee angle was 90°, and the ankle axis of rotation coincided with axis of the motor. To avoid any variation in body temperature, the rectal temperature was monitored and computed by a temperature controller (ATC 1000, World Precision Instruments, United States) that adjusted the temperature of the heating plate to maintain the rectal temperature at 37°C. Stimulation of the rat ankle plantarflexors was done percutaneously via Ag/AgCl surface electrodes (StimCom TS0020, Comepa, France). A constant-current electrical stimulator (DS 7A, Digitimer, United Kingdom) was used to deliver square waves (pulse width = 1 ms). The stimulator was triggered and controlled with automated scripts by an A/D board (PowerLab 8/35, ADInstruments, Australia) and associated software (LabChart 7.3, ADInstruments, Australia).

The protocol consisted in the evaluation of the maximal torque from the torque-frequency relationship in isometric condition and in the evaluation of maximal power from the torque-velocity relationship in concentric condition.

To determine the maximal torque from the torque-frequency curve, the ankle angle was set at 90° and the plantar flexors stimulated at frequencies corresponding to the upper plateau, i.e., from 100 to 175 Hz (100, 125, 150, and 175 Hz). These contractions were 200 ms in length with 45 s between contractions, and done in order of increasing stimulation frequency. The reported isometric torque values were calculated as the peak isometric torque minus resting torque. The maximal isometric torque was determined from the torque responses to the different stimulation frequencies.

After 3 min of rest, the torque-velocity curve was determined from 4 concentric contractions realized at angular velocities of 300–700°/s in old rats and 400–800°/s in young rats (i.e., 300, 400, 500, 600, 700, and 800°/s), realized in order of decreasing velocity. The maximal power is evoked at these velocity ranges in old and young rats (Verney et al., 2017). These contractions were evoked over a 40°-angular amplitude, centered about the 90° ankle angle (i.e., from 70° to 110°). This movement range was chosen because it coincides closely to that of the ankle during the stance phase of voluntary ambulation (i.e., from 72° to 111°; Rossi et al., 2020). The contractions were evoked every 45 s by stimulation trains delivered at 175 Hz for only the duration necessary to complete the movement; the 175-Hz frequency was used according to Warren et al. Cacho et al., 2008 recommendation, as the frequency yielding maximal isometric tetanic torque. A power-velocity curve was then computed from this torque-velocity curve. The maximal concentric power was determined from the power outputs at the different angular velocities. In the current experiment, only the highest stimulation frequencies were used since the aim was to determine the maximal torque value. This maximal torque value was used for subsequent data analysis.

### Statistical Analysis

Data are presented as means ± SD. Data analyses were performed using IBM SPSS Statistics, version 27 (IBM Corp., Armonk, NY, United States). Distributions of continuous variables were examined for skewness and kurtosis, and were logarithmically transformed when appropriate. Log-transformed variables are presented as untransformed values for ease of reading (i.e., insulin, glucose, FGIR, and intramuscular TAGs). A two-way analysis of variance (ANOVA) was performed to test the effect of the experimental nutritional conditions and the effect of age. Pairwise multiple comparisons were performed applying the Bonferroni correction. Spearman’s correlation was used to examine the relationship between outcome variables and other variables selected *a priori* as potential confounders (i.e., myosteatosis and insulin resistance). Since age groups differed in baseline absolute values, the observed changes were scaled to the baseline values and expressed as delta changes/variations expressed as percentage. The level of significance for all statistical tests was set at *p* < 0.05.

## Results

Despite diets providing higher energy were administered to the HF12 and HF25 groups compared to the two ST groups, rats in the high-fat diet groups self-limited their food intake, and no significant differences were observed in calories ingested (energy intake and food intake are shown in the Supplementary Figure 1). Hence, the following findings are mainly based on the effects of macronutrient manipulation.

### Body Weight and Body Composition

**FIGURE 1:**
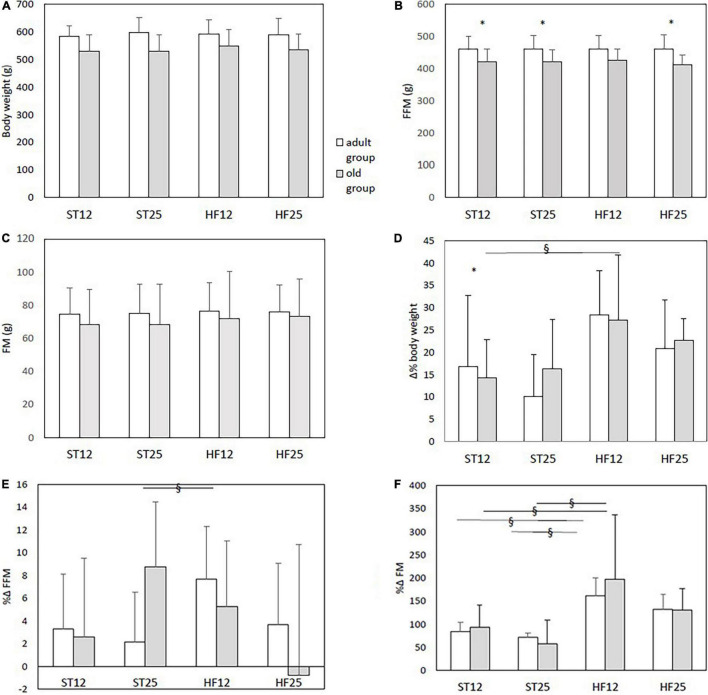
**(A–C)** Body weight, fat-free mass (FFM), and fat mass (FM) at baseline. **(D)** %Δ body weight. Diet main effect, *p* = 0.004; age group main effect, *p* = 0.044; diet × age group interaction, (*p* = n.s.). § diet effect (same age) in the old group: ST12 vs. HF12, *p* = 0.039; * age effect (same diet) in ST12 rats, *p* = 0.013. **(E)** %Δ fat-free mass (FFM): § diet effect (same age) in the old group ST25 vs. ST12, *p* = 0.048. **(F)** %Δ fat mass (FM): diet main effect, *p* < 0.001; age group main effect, n.s.. § diet effect (same age) in the adult group: HF12 vs. ST12, *p* = 0.007; HF12 vs. ST25, *p* = 0.007. § diet effect (same age) in the old group HF12 vs. ST12, *p* < 0.001, and HF12 vs. ST25, *p* < 0.001. Adult rats: ST12 (*n* = 12); ST25 (*n* = 12); HF12 (*n* = 12); HF25 (*n* = 11). Old rats: ST12 (*n* = 11); ST25 (*n* = 11); HF12 (*n* = 9); HF25 (*n* = 7).

Baseline data and percent delta changes from baseline in body weight and body composition are displayed in Figures 1A–F. Old rats in the HF12 group exhibited a higher body weight increase compared to their old counterparts in the ST12 group (diet effect, same age, *p* = 0.039), and a similar tendency was observed in adult rats in the HF12 group compared with adult rats in the ST12 group (diet effect, same age, *p* = 0.058). Concerning FFM, just old rats in the ST25 group had a larger – tough marginally significant – percent increase from baseline compared with the old ST12 group (diet effect, same age, *p* = 0.048), and also tended to have a greater increase of FFM than adult ST25 rats (age effect, same diet, *p* = 0.061), see Figure 1E. Changes in total adiposity are displayed in Figure 1F: the adult HF12 group exhibited a higher percent increase of FM compared to the ST12 and the ST25 groups (HF12 vs. ST12: *p* = 0.007; HF12 vs. ST25: *p* = 0.007); the FM percent augmentation tended to be higher in the adult HF25 group than the adults ST25 group (*p* = 0.070) and ST12 group (*p* = 0.086). In the old group a significant diet effect (same age) emerged for the percent delta increase of FM, higher in the HF12 group than ST12 group (*p* < 0.001) and than the ST25 group (*p* < 0.001); only a tendency was observed for a higher percent increase from baseline in the HF25 group compared to the ST25 group (*p* = 0.080). The tibialis anterior muscle weight has been provided in the Supplementary Figure 2, with a clear-cut age effect in all groups (*p* < 0.001).

### Glucose Homeostasis and Insulin Resistance

Results are displayed in the Supplementary Figures 3A–C. Plasma glucose levels were significantly lower in old HF25 rats compared to the old HF12 rats (diet effect, *p* = 0.008), old ST25 rats (diet effect, *p* = 0.003), and old ST12 rats (diet effect, *p* < 0.001). In addition, old rats in the ST12 (age effect, same diet, *p* = 0.002), ST25 (age effect, same diet, *p* = 0.049), and HF12 (age effect, same diet, *p* = 0.015) group had higher glucose levels than their adult counterparts. Insulin levels tended to be higher in the adult HF12 group compared with the adult ST12 group (diet effect, same age, *p* = 0.051). Old HF12 rats had lower insulin levels than their adult counterparts (age effect, same diet, *p* = 0.039). Similarly old HF25 rats tended to have lower insulin concentrations than adult HF25 rats (*p* = 0.071). Fasting glucose insulin ratio (FGIR) was higher in old rats in the HF25 compared to adult rats (age effect, *p* = 0.041), HF12 (*p* = 0.012), and ST25 (age effect *p* < 0.001) groups compared to their adult counterparts.

### Ectopic Lipid Content (Intramuscular Lipids)

**FIGURE 2:**
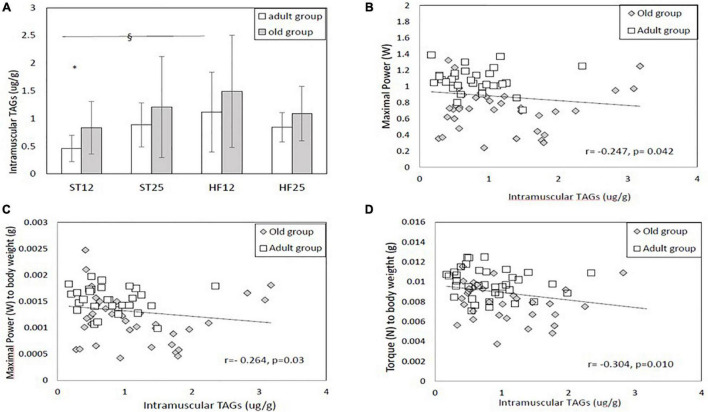
**(A)** Intramuscular triacylglycerols (TAGs). § Diet effect (same age) in the adult group, HF12 vs. ST12, *p* = 0.022. *Age effect (same diet): ST12 groups, *p* = 0.030. Adult rats: ST12 (*n* = 12); ST25 (*n* = 12); HF12 (*n* = 12); HF25 (*n* = 11). Old rats: ST12 (*n* = 11); ST25 (*n* = 11); HF12 (*n* = 9); HF25 (*n* = 7). **(B–D)** Correlations between intramuscular TAGs and maximal power, maximal power to body weight ratio, and maximal isometric torque to body weight ratio.

As shown in Figure 2A, adult rats in the HFD12 group exhibited higher intramuscular triacylglycerols (TAGs) in tibialis anterior muscle than the control ST12 group (HF12 vs. ST12: diet effect, same age, *p* = 0.022). Adult HF25 rats tended to have higher intramuscular TAGs than adult ST12 rats (diet effect, same age, *p* = 0.10). Intramuscular TAGs were significantly higher in old ST12 rats than adult ST12 rats (age effect, same diet, *p* = 0.030).

### Muscle Functional Properties

#### Maximal Isometric Torque

**FIGURE 3:**
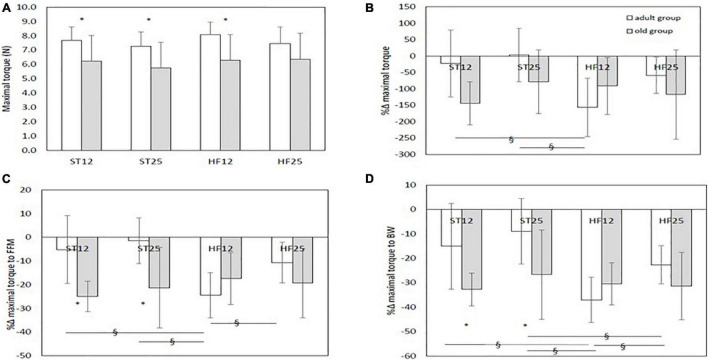
**(A)** Maximal isometric torque at baseline.*Age effect: *p* = 0.003 (ST12); *p* = 0.004 (ST25); *p* = 0.005 (HF12). **(B)** %Δ maximal isometric torque. Diet main effect: *p* = 0.084; age group main effect; *p* < 0.001; diet × age group interaction: *p* = 0.014. § Diet effect (same age) in the adult group, *p* = 0.021 (ST12 vs. HF12) and *p* = 0.001 (ST25 vs. HF12). *Age effect (same diet), *p* < 0.001 (ST12) and *p* = 0.003 (ST25). **(C)** %Δ maximal isometric torque to fat-free mass (FFM) ratio: diet main effect, *p* = 0.085; age group main effect, *p* < 0.001; diet × age group interaction, *p* = 0.001. § Diet effect (same age) in the adult group, *p* = 0.001 (ST12 vs. HF12); *p* < 0.001 (ST25 vs. HF12); *p* = 0.027 (HF12 vs. HF25). *Age effect (same diet): *p* < 0.001 (ST12); *p* < 0.001 (ST25). **(D)** %Δ maximal isometric torque to body weight (BW) ratio: diet main effect, *p* = 0.002; age group main effect, *p* = 0.001; diet × age group interaction, *p* = 0.011. § Diet effect (same age) in the adult group, *p* < 0.001 (ST12 vs. HF12); *p* < 0.001 (ST25 vs. HF12); *p* = 0.045 (ST25 vs. HF25); *p* = 0.039 (HF12 vs. HF25). *Age effect (same diet): *p* = 0.002 (ST12); *p* = 0.002 (ST25). Adult rats: ST12 (*n* = 12); ST25 (*n* = 12); HF12 (*n* = 12); HF25 (*n* = 11). Old rats: ST12 (*n* = 11); ST25 (*n* = 11); HF12 (*n* = 9); HF25 (*n* = 7).

Baseline values of maximal isometric torque, with old rats in the ST12, ST25, and HF12 groups exhibiting significant lower values than their adult counterparts (*p* = 0.003, *p* = 0.004, and *p* = 0.005, respectively, as displayed in Figures 3A,B shows the percent delta changes from baseline in maximal isometric torque: a more pronounced decrease in maximal isometric torque was described in adult rats in the HF12 group than the adult ST12 group (diet effect, same age, *p* = 0.021). Adults HF12 rats also reported a significant negative change when compared to the ST25 group, in which maximal isometric torqued modestly increased (diet effect, same age, *p* = 0.001). A significant larger reduction from baseline was found in old ST12 and ST25 rats compared to their adult counterparts (age effect, same diet, *p* < 0.001 an *p* = 0.003, respectively). No significant differences were detected between old rats fed either a ST or HF diet.

Relative maximal isometric torque as normalized to FFM, as shown in Figure 3C, decreased more in the adult HF12 group than the ST12 (diet effect, same age, *p* = 0.001), ST25 (diet effect, same age, *p* < 0.001), and HF25 (diet effect, same age, *p* = 0.027) adult groups. A larger decrease from baseline was also found in old rats in the ST12 and ST25 groups than their adult counterparts (both age effects, same diet, *p* < 0.001, respectively). No significant differences were detected between old rats fed either a ST or HF diet. As shown in Figure 3D, variations in relative isometric torque as normalized to body weight were larger in the adult HF12 rats than the ST12 diet effect, same age, *p* < 0.001), ST25 (diet effect, same age, *p* < 0.001) and HF25 rats (diet effect, same age, *p* = 0.039). Adult rats in the HF25 group had a larger decrease from baseline compared with adult rats in the ST25 group (diet effect, same age, *p* = 0.045). A significantly greater decrease was also observed in old rats in the ST12 and ST25 groups compared to their adult counterparts (both age effects, same diet: *p* = 0.002, respectively). No significant differences were detected between old rats fed either a STD or HFD diet.

#### Maximal Power

**FIGURE 4:**
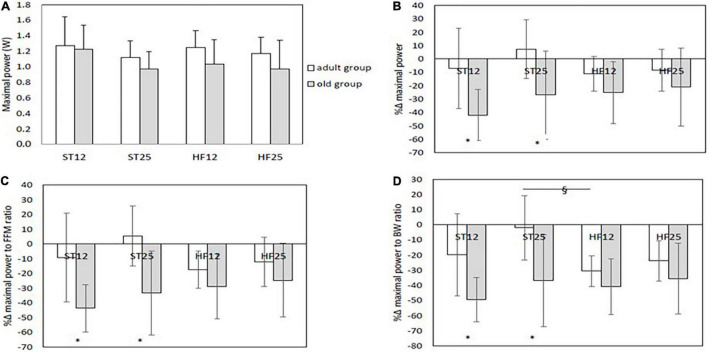
**(A)** Maximal power at baseline. **(B)** %Δ maximal power: *age effect (same diet) *p* < 0.001 (ST12) and *p* < 0.001 (ST25). **(C)** %Δ maximal power to fat-free mass (FFM) ratio: *age effect (same diet) *p* < 0.001 (ST12) and *p* < 0.001 (ST25). **(D)** %Δ maximal power to body weight (BW) ratio: § diet effect (same age) in the adult group, ST25 vs. HF12, *p* = 0.007. *Age effect (same diet): *p* = 0.001 (ST12) and *p* < 0.001 (ST25). Adult rats: ST12 (*n* = 12); ST25 (*n* = 12); HF12 (*n* = 12); HF25 (*n* = 11). Old rats: ST12 (*n* = 11); ST25 (*n* = 11); HF12 (*n* = 9); HF25 (*n* = 7).

Maximal power at baseline is displayed in Figure 4A: just a tendency toward lower power value was detected in old HF12 rats compared to their adult counterparts (*p* = 0.09). When considering maximal power relative changes from baseline, as depicted in Figure 4B, old rats in the ST12 and in the ST25 groups reported a more marked decrease from baseline compared to their adult counterparts (both age effects, same diet: *p* < 0.001, respectively). Similar observations were found as **percent delta changes in maximal power normalized to FFM** (Figure 4C). In this case a tendency emerged for a diet effect in the adult group, with a larger (negative) percent change in the HF12 group than the ST25 group (positive percent change), *p* = 0.076. **Maximal power was also normalized to body weight**. For this outcome variable, percent delta changes are summarized in Figure 4D. In the adult group percent delta change in the ST25 group was significantly lower than the HF12 group (diet effect, same age, *p* = 0.007), and tended to be lower than the HF25 group (diet effect, same age, *p* = 0.083). In addition, old rats in the ST12 group and the ST25 group exhibited a more marked percent delta decrease than their adult counterparts (age effect, same age, *p* = 0.001 and *p* < 0.001, respectively). Whatever, the expression of maximal power (in absolute or percent delta changes), no significant differences were detected between old rats fed either a STD or HFD diet. As Figures 2B–D show, a significant negative relationship emerged between intramuscular TAGs and maximal and relative power (normalized to body weight) (Spearman Correlation coefficients: *r* = −0.247, *p* = 0.042, and *r* = −0.264, *p* = 0.03, respectively). Also relative maximal isometric torque (i.e., normalized to body weight) was significantly inversely correlated with intramuscular TAGs (Spearman Correlation coefficient, *r* = −0.304, *p* = 0.010). Maximal power and relative power (i. e. normalized to FFM) were negatively correlated with FGIR (Spearman Correlation coefficients, *r* = −0.383, *p* < 0.001, and *r* = −0.250, *p* = 0.025, respectively). Maximal isometric torque and relative isometric torque (normalized to FFM) were negatively correlated with FGIR (Spearman Correlation coefficients: *r* = −0.327, *p* = 0.004, and *r* = −0.290, *p* = 0.011, respectively).

## Discussion

The majority of data concerning sarcopenic and dynapenic obesity rely on findings from clinical studies (Poggiogalle et al., 2014; Scott et al., 2014; Rossi et al., 2017, 2020), and evidence is scarce in terms of underlying mechanisms. The present study conducted in rats sheds light on the potential interaction among aging and nutrient imbalance in the development of the phenotype of sarcopenic/dynapenic obesity. Based on the present findings, interestingly, in adult rats high-protein intake combined with high-fat diet determined a lower decrease in relative isometric torque, normalized to either FFM or body weight, compared with adult rats fed a high-fat normal-protein diet. When considering maximal isometric torque (not scaled to body weight/body composition), in adult rats a high-fat standard- protein diet (HF12) elicited a more pronounced decrease in maximal isometric torque values than a normal- fat standard diet, irrespective of protein content. Notably, in adult rats, high-protein diet, when combined with normal fat intake, was able to prevent significant changes in maximal isometric torque values, allowing even a slight increase than their counterparts fed a high-fat normal-protein (HF12) diet. In terms of muscle power, indeed high-fat diet resulted to be detrimental to relative muscle power, as normalized to body weight, that decreased to a larger extent in adult rats fed a high-fat normal-protein diet (and tended to be lower in adult rats fed a high-fat high- protein diet) than their counterparts fed a normal-fat, high-protein diet. However, the diet-related effects on muscle power were less evident than for isometric torque.

### Intramuscular Lipid Infiltration

Based on our observations in terms of power and isometric torque, the proportion of dietary protein may modulate the response to high-fat diet in terms of force generation. The effect of HFD observed in adults, with the enhanced protein intake (25%) conferring some kind of protection against the detrimental effects of HFD, may be linked to the reduced intramuscular fat in this group, which may have contributed to preserve, at least partly, the contractile properties. Intramuscular lipid infiltration was more pronounced in adult rats fed a high-fat normal protein diet than their adult counterparts fed a ST12 diet. Conversely, intramuscular TAGs were not increased in the HFD25 groups, and a potential role for high-protein diet in preventing ectopic lipid deposition needs to be explored in future research. However, our observations are somewhat in line with recent findings from French et al. (2017), showing that Zucker lean and obese rats fed a high-protein diet (40% protein) exhibited lower lipid deposition in the skeletal muscle than lean and obese rats on a 20% protein diet. Those findings were likely to be explained by a lower expression of fatty acid synthase and PPARδ (French et al., 2017). This suggests a role of dietary protein to reduce gene expression related to fat deposition in skeletal muscle. Similar observations were reported in a study by Chaumontet et al. (2015), demonstrating a reduction of lipogenesis in adult male Wistar rats fed a high-protein diet, through the decrease of mRNA expression of fatty acid synthase, acetyl-coA carboxylase and a and b and sterol regulatory element binding transcription factor 1c. Also Pichon et al. (2006) showed that hepatic lipogenesis and fat deposition in adipose tissue diminished after exposure to high-protein diet (55%).

Interestingly, we found a negative relationship between intramuscular TAGs and maximal and relative power (normalized to body weight), and relative maximal isometric torque (i.e., normalized to body weight). This has already been reported in non-obese elderly (Sipila and Suominen, 1994). Among the potential explanations, a negative effect of intramuscular fat on maximal nervous activation level has been postulated (Yoshida et al., 2012). In the current study this can be ruled out since the contractions were evoked electrically at optimal stimulation intensity. An alternative explanation is represented by the adverse effects of intramuscular fat on muscle contractile mechanics. Indeed, a recent modeling study (Rahemi et al., 2015) predicted that intramuscular fat infiltration could negatively affect muscle strength, by limiting muscle fiber shortening and transverse deformation of the skeletal muscle during contraction.

### Skeletal Muscle Functional Properties and Obesity

Our observations underscore the complexity of muscle functional assessment in the context of sarcopenic obesity, with emphasis on the body size effect on strength capacity; ratio scaling, based on dividing the muscle performance outcomes by body mass (or FFM), is a common practice to account for the body size effect on performance variables (Crewther et al., 2011). Nonetheless, the assumption for ratio scaling is the linear relationship between body mass and performance, and further research is needed to optimize an allometric scaling approach especially for translational purposes in aging individuals with the coexistence of age-related sarcopenia and obesity (McGrath, 2019). Investigations focusing on the effects of obesity on muscle function have described that obese people elicited higher absolute muscle strength and power than normal-weight individuals (Hulens et al., 2001; Lafortuna et al., 2005; Maffiuletti et al., 2007). Coping chronically with the overload associated with high levels of fat mass occurring in obese individuals might be compared to a chronic stimulus for the loaded neuromuscular system. Bosco et al. (1986) reported increases in muscle power in anti-gravity muscles in normal weight subjects wearing a weighted vest during 3 weeks to stimulate hypergravity. This extra-loading might be assimilated to resistance exercise. However, due to the gradual and sustained increases in fat mass that an obese individual would experience, the adaptations to skeletal muscle to body weight excess may differ from that of a healthy individual undertaking loaded resistance exercise, notably in relation with co-morbidities that can be associated with obesity. As such, there exists experimental evidence that young “healthy” obese adolescents display positive neuromuscular adaptations to obesity, both in quantitative (greater muscle mass) and qualitative terms (higher pennation angle, improved maximal nervous activation level of the lower limb muscles) (Garcia-Vicencio et al., 2016). However, these positive adaptations are much less obvious in older adult obese (Tomlinson et al., 2014a,b), who have developed the adverse co-morbidities of obesity (low-grade inflammation, insulin resistance, and myosteatosis) that may adversely affect neuromuscular properties. Should any positive adaptations occur in response to chronic overloading, this is never sufficient from a functional point. Indeed, if torque or power per unit muscle surface/volume may be higher in obese (Maffiuletti et al., 2013), strength and power per unit body weight is always lower in obese, thereby underlying the functional difficulties encountered by obese individuals in daily-living activities requiring the displacement of body mass (Hulens et al., 2001; Abdelmoula et al., 2012; Maffiuletti et al., 2013; Bollinger, 2017). In this study, old rats fed a standard diet with either normal or high- protein, but not old rats fed high-fat diets, reported a more marked decrease of muscle performance outcomes compared to their adult counterparts, with analogous trajectories as changes in both maximal and relative isometric torque and power, respectively. One can hypothesize that in the late phase of aging a high-energy diet (though it comes from fat mainly as in the present study) can enhance protein intake utilization (Swanson, 1951; Edens et al., 1986) due to a potential beneficial effect of non-protein calories on protein metabolism, and prevent significant performance impairment irrespective of normal or high protein intake in the elderly. When it comes to the less clear-cut effects of high-fat diet in old rats than adult rats, it is difficult to assess to which extent skeletal muscle fat infiltration as well as muscle performance impairment related to high-fat feeding can be disguised by the interference of aging, especially in our old rats representing extreme late life conditions (Marcus et al., 2010). In rodents, only a limited number of studies have examined the combined effects of aging and high-fat diet on skeletal muscle function (Abrigo et al., 2016; Bott et al., 2017; Hill et al., 2019; Eshima et al., 2020). Evidence indicates that high-fat diet (HFD) consumption exacerbates the age-related decline in muscle function. However, methodological approaches, age of mice, high-fat diet duration and contractile mode-specific response are largely variable in prior studies and make difficult to identify a specific effect. After 9 weeks of HFD in 79-week-old mice, no change in isometric force and absolute power were found in locomotor muscles (Hill et al., 2019). By contrast, muscle quality of old obese mice diaphragm was significantly lower compared to lean, age-matched counterparts. Eshima et al. (2020) showed that chronic exposure to high-fat diet (i.e., 20 months) exacerbated the impairment of the skeletal muscle contractile force due to aging and, at least in part, to dysfunction in the intracellular Ca^2+^ release. The authors also observed a higher intramyocellular lipid content in extensor digitorum longus which may be a marker for impaired muscle contractile force caused by aging and HFD.

### Body Weight and Body Composition

Given that rats receiving high-calorie diets self-limited their energy intake, leading to a lack of differences in terms of calorie intake among the four groups, observations can be interpreted as effects of macronutrient manipulation. Our findings with regard to the self-limitation of ingested calories are in line with extant data exploring adaptation to high- protein diet in rodents (Pichon et al., 2006). Solon-Biet et al. have shown that in mice food intake was reduced once protein intake exceeded about 10 kJ/day, whereas carbohydrate intake decelerated once carbohydrate intake exceeded about 15 kJ/day (Solon-Biet et al., 2020). Indeed based on animal and human studies, it is well-established that protein intake acts as a pivotal modulator of mechanisms of appetite, with elevated protein intakes associated with high satiety and satiation (Fromentin et al., 2012; Martens and Westerterp-Plantenga, 2014; Carreiro et al., 2016). Nonetheless rats in the high-fat groups reported an increase in body weight and in adiposity even in the absence of energy excess with respect to the two standard diets groups. These data are in agreement with prior animal studies revealing that an isocaloric high-fat diet induced weight gain and fat accumulation than the isocaloric control diet (Boqué et al., 2009; Hariri and Thibault, 2010; Lomba et al., 2010). The relevant role of dietary composition, especially in terms of high-fat and high-sucrose diets (Boqué et al., 2009; Lomba et al., 2010), emerged as an important determinant of body weight regulation, yet the interplay between lipid and protein has been poorly investigated. From our findings, in old and adult rats high-fat diet with normal protein, but not a high-fat diet combined with high-protein, determined a larger increase in body weight than their counterparts fed a standard diet (isocaloric and normal protein diet). When considering body composition, both old and adult HF12 groups exhibited a larger increase of total adiposity compared to the standard-diet counterparts. Fat mass augmentation just tended to be higher in the old and adult HF25 groups than their ST25 counterparts. Concerning the lean compartment, old rats in the ST25 group had a larger increase from baseline compared with the old ST12 group, suggesting that high-fat feeding limited somewhat fat-free mass expansion regardless of protein intake. These observations may be related to the presence of anabolic resistance in the aged rats (Tardif et al., 2014), as well as in aged individuals (Boirie et al., 2014; Ten Haaf et al., 2018) that is further exacerbated by the occurrence of obesity. Tardif et al. demonstrated that diet-induced obesity (DIO) impaired muscle protein synthesis in old male Wistar rats, inducing a ≈18–23% decrease of muscle total and mitochondrial protein synthesis rate in DIO old rats compared with control old rats (Tardif et al., 2014). Furthermore, a downregulation of genes involved in lipogenesis and an up-regulation of genes related to fatty acid β-oxidation have been described after high-protein diets (Akieda-Asai et al., 2013; Chaumontet et al., 2015). More recently, an intriguing non-leptin dependent model has been hypothesized to support a mechanism preventing weight gain in response to high-fat and/or high-calorie intake in order to defend the body from further gain in weight and adipose depots (Ravussin et al., 2014, 2018). In this original homeostatic scenario, the role of macronutrient interaction, especially the potential role played by different protein amounts, and the effect of aging have yet to be established. In fact, based on our observations, one can hypothesize that a threshold of protein intake can be responsible for anabolic or catabolic response in the presence of high-fat feeding. In addition, an insufficient stimulation of insulin secretion in relation to carbohydrate dietary content (Guillet and Boirie, 2005), as well as anabolic resistance to insulin in the old groups (Boirie, 2003), can be postulated to explain body weight and fat-free mass trajectories over the 10-week dietary intervention in rats ingesting high-fat high-protein, and relatively low-carbohydrate, diet.

### Glucose Homeostasis, Insulin Resistance, and Skeletal Muscle Contractile Properties

Alterations in glucose metabolism and insulin resistance represent additional explanations for our observations, with relevant impact on protein anabolism and skeletal muscle functional properties. In the present study maximal power and relative power (i. e. normalized to FFM), maximal isometric torque and relative isometric torque (i.e., normalized to FFM) resulted to be significantly negatively correlated with insulin resistance. Insulin is a crucial factor in the regulation of protein anabolism and protein breakdown (Proud, 2006). In previous studies, insulin resistance has also been shown to influence contractile properties (Orlando et al., 2016; Poggiogalle et al., 2019). Specifically, insulin resistance is also able to disturb the Na^+^/K^+^ pump activity and the excitation-contraction coupling (Orlando et al., 2016). Altogether these factors may account for the qualitative alterations of torque and power observed in the current study. Another potential explanation for our findings in terms of dynapenic phenotype may be related to elevated glucose levels especially in the old groups. Extensive evidence from cellular, clinical, and animal studies supports a detrimental effect of hyperglycemia on muscle quality and muscle strength due to the interference of acute as well as long-term elevated blood glucose levels on Ca^2+^ signaling, skeletal muscle excitability, and contractility (Kalyani et al., 2015; Yoon et al., 2016; Hernández-Ochoa et al., 2017). In the current study, some results differed depending on whether torque or power data was considered. Maximal isometric torque (or force) is frequently reported in experimental studies. From a functional point of view, maximal power is more relevant since (i) isometric contractions are very rare in spontaneous locomotion and (ii) maximal torque is rarely produced during locomotion. As power results from the product of torque by contraction velocity, this also gives additional insights into muscle function. Maximal evoked torque is only dependent on muscle mass, specific tension and sarcolemmal excitability. Any alterations of contraction velocity do not affect torque production. Overall, the results were more obvious for torque than power. This can be explained by the fact that the beneficial effect of protein supplementation mainly impacts torque production, possibly via beneficial effects on muscle mass and specific tension. The high-fat diet can have detrimental effects on muscle protein synthesis (and thus on muscle mass and specific tension), but also on contractile velocity (Tallis et al., 2017). However, the effects on contractile velocity are less likely to be counteracted by protein supplementation. This certainly explains why the beneficial effects of protein supplementation are less obvious on power than torque in the context of a high-fat diet feeding.

As differences between young and old counterparts are commonly explored in aging research (Smeuninx et al., 2017), a point of strength of the present study is represented by the comparison of an intermediate phase of aging, that is adult age, with the more advanced stage of late life conditions, providing insights on transitional aspects of lifespan changes in the response to nutritional modulation of body composition and physical functionality. However, as a limitation to the present study, data concerning the level of physical activity in the calorimetric cage are lacking, since no measurement of physical activity of the animals was realized. Rats were housed in individual cages without any enrichment, hence the possibility for the ST12 group to have a higher physical activity level than the HF12 group is unlikely, in line with evidence not supporting a response in terms of spontaneous locomotor activity in rodents fed a high-fat diet (Brownlow et al., 1996; Wu et al., 2017; Stojakovic et al., 2018), and behavioral changes induced by high-fat diet resulted independent of overall locomotion (Deal et al., 2020). However, other authors observed an increase in spontaneous physical activity in the dark cycle in rats fed a high-fat diet, but those changes counterbalanced the adaptive thermogenetic response in the brown adipose tissue, offsetting the impact of physical activity on total daily energy expenditure (So et al., 2011). Thus the higher energy density of the diet provided to the rats and the higher proportion of fat and the difference of the types of fat ingested are the major determinants of differences in terms of weight gain (So et al., 2011). The lack of investigation of genetic determinants of the outcome measures (i.e., skeletal muscle strength and power) and of the metabolic flux during the contraction (Shortreed et al., 2009; Roy et al., 2016) represents another limitation, needing to be addressed in future studies based on mechanistic experiments focusing on the contribution of muscle lipid metabolism (rather than just lipid infiltration as in the present study) to weakness. Finally, the present study did not focus on the potential influence of inflammation on muscle contractility (Laurentius et al., 2019).

## Conclusion

Detrimental effects of high-fat diet on skeletal muscle performance is mitigated by high-protein intake in adult rats but not in old rats. In isocaloric conditions, higher protein intake modulated muscle lipid infiltration, but did not improve age-related changes in body compartments in old rats fed a high-fat diet. Further research is warranted to identify nutritional strategies to improve body composition and physical functionality in the aging population.

## Data Availability Statement

The original contributions presented in the study are included in the article/Supplementary Material, further inquiries can be directed to the corresponding author/s.

## Ethics Statement

The animal study was reviewed and approved by the Ethics Committee Clermont Auvergne University, INRA, UNH, CRNH Auvergne, Clermont-Ferrand, France.

## Author Contributions

CGu, YB, and SW contributed to the conception and design of the study. AC, FC, J-PR, SD, OL-B, JS, CGi, VP, PL, and EP conducted the experiments. CGu, EP, and FR organized the database. EP performed the statistical analysis. EP and CGu wrote the first draft of the manuscript. CGu, EP, and VM wrote the sections of the manuscript. All authors contributed to manuscript revision, read, and approved the submitted version.

## Conflict of Interest

The authors declare that the research was conducted in the absence of any commercial or financial relationships that could be construed as a potential conflict of interest.

## Publisher’s Note

All claims expressed in this article are solely those of the authors and do not necessarily represent those of their affiliated organizations, or those of the publisher, the editors and the reviewers. Any product that may be evaluated in this article, or claim that may be made by its manufacturer, is not guaranteed or endorsed by the publisher.
