# Supplementary figures and images for: Deleterious Effect of High-Fat Diet on Skeletal Muscle Performance Is Prevented by High-Protein Intake in Adult Rats but Not in Old Rats
Source: Front Physiol. 2022 Jan 17;12:749049. doi: 10.3389/fphys.2021.749049 (PMC8801536; doi:10.3389/fphys.2021.749049)

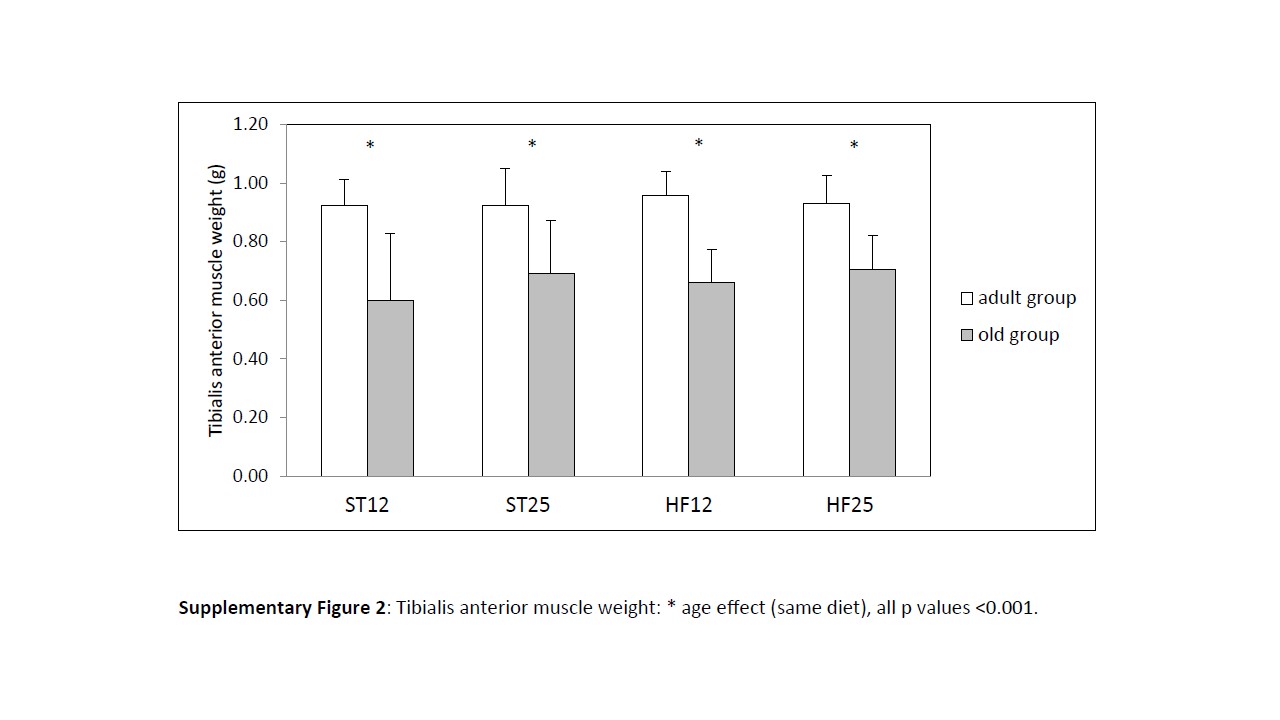

Supplement: Supplementary file 1 [file Image_1.JPEG]

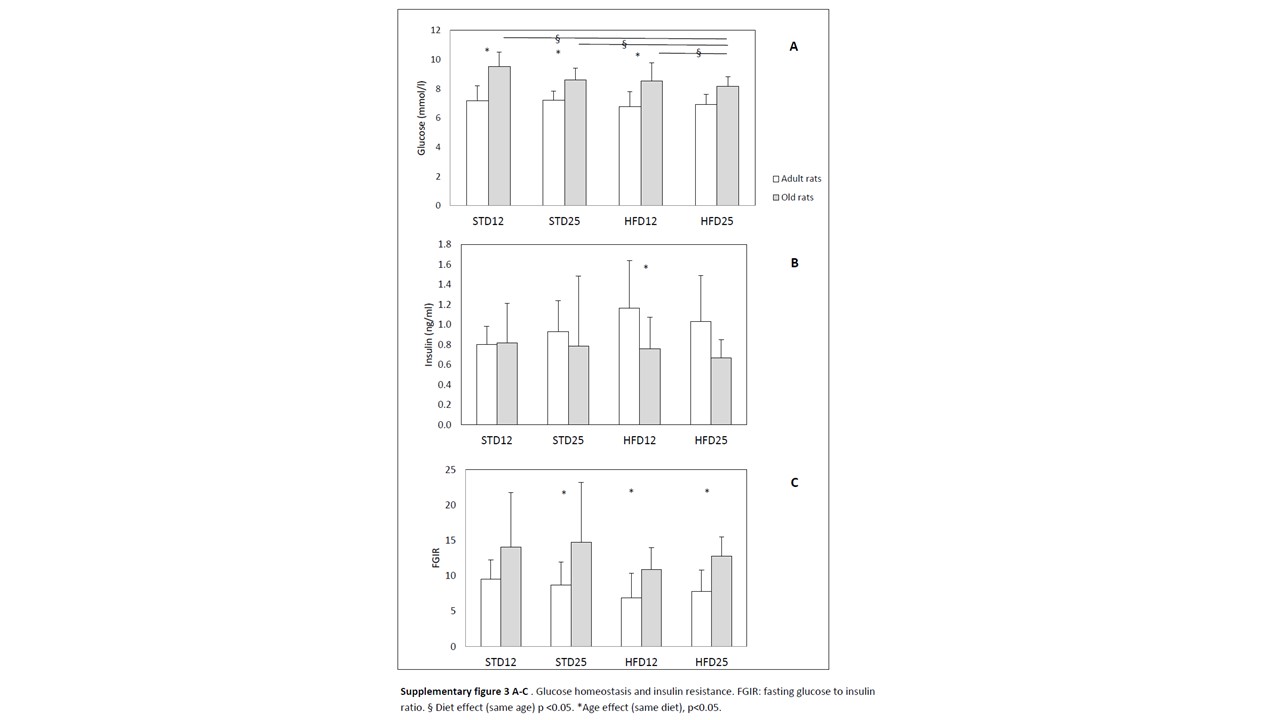

Supplement: Supplementary file 2 [file Image_2.JPEG]

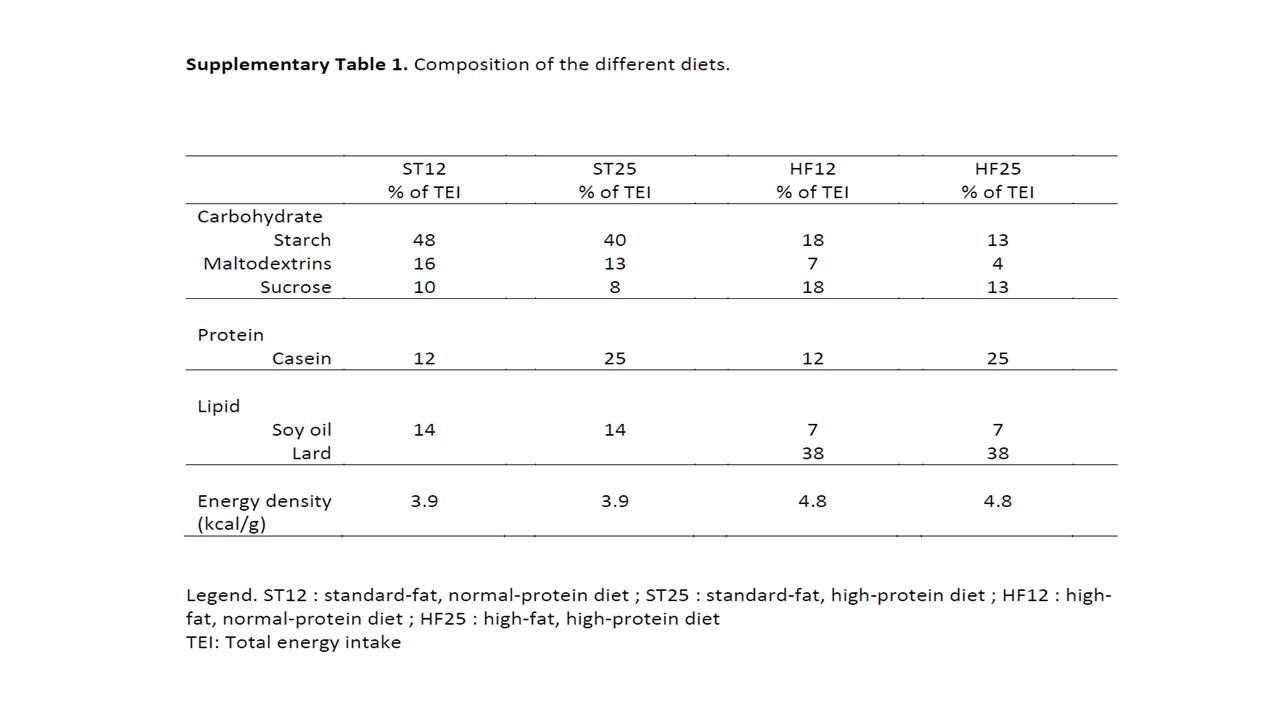

Supplement: Supplementary file 3 [file Image_3.JPEG]

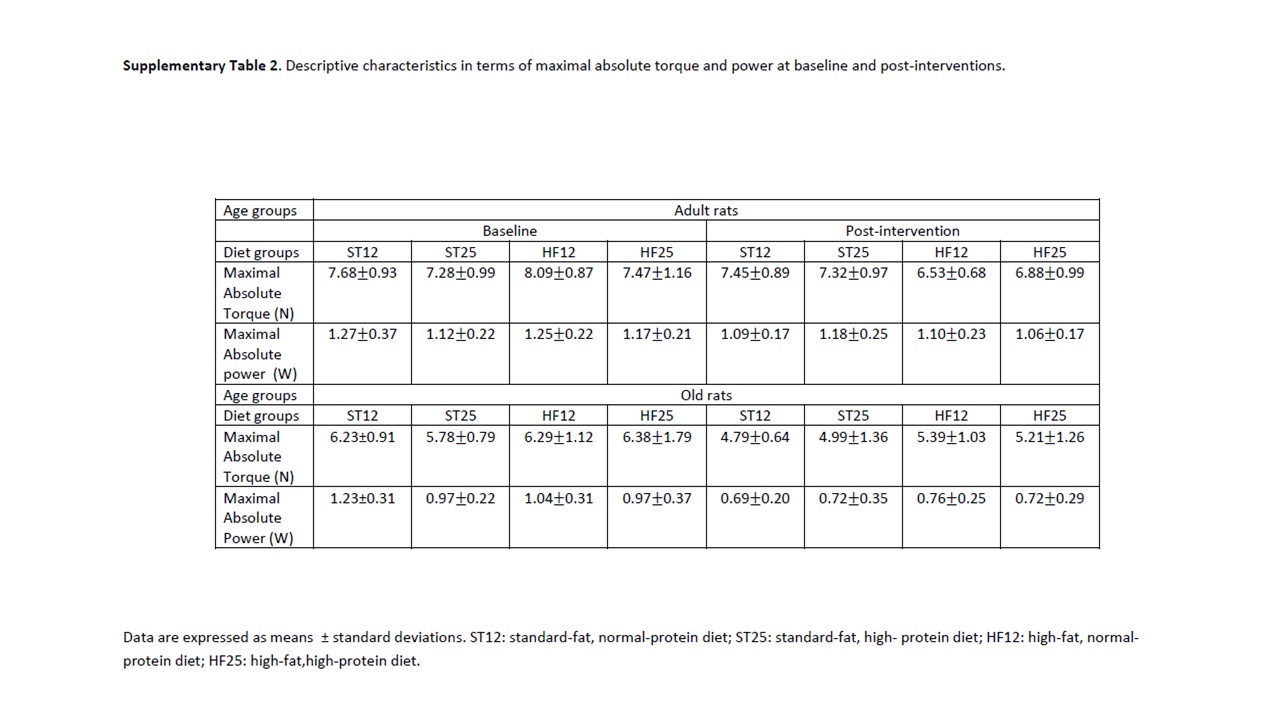

Supplement: Supplementary file 4 [file Image_4.JPEG]

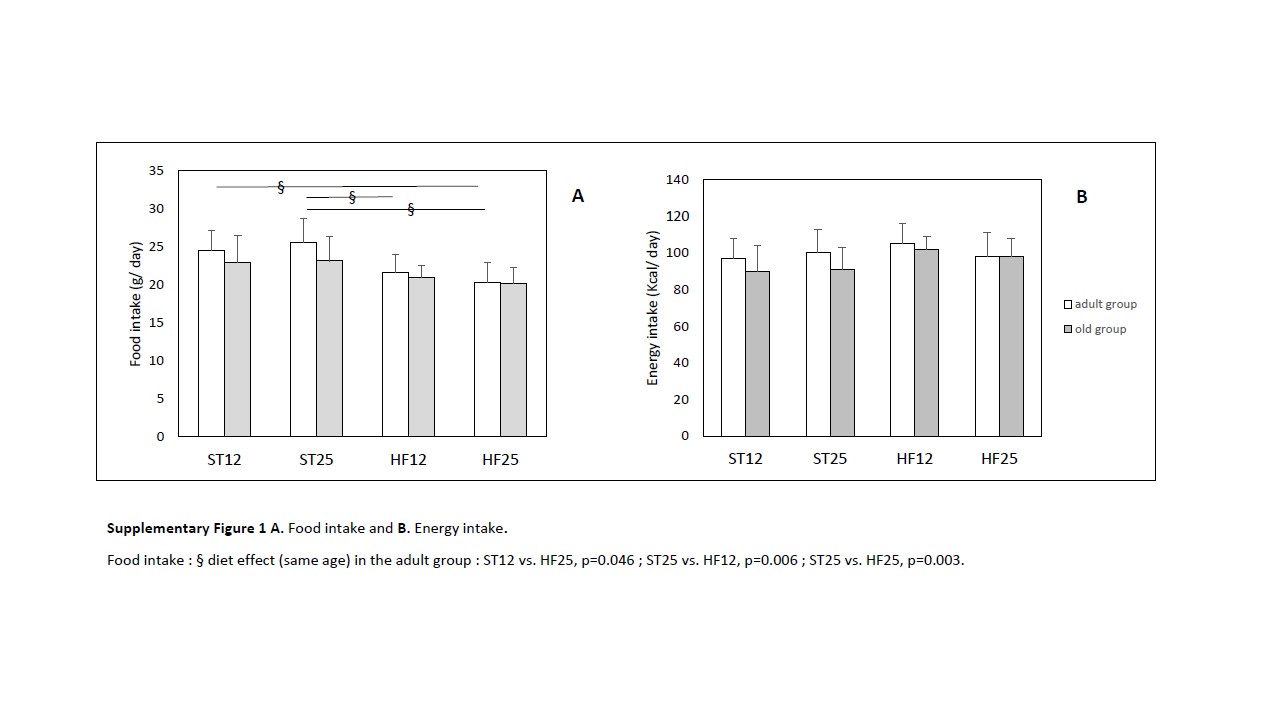

Supplement: Supplementary file 5 [file Image_5.JPEG]
